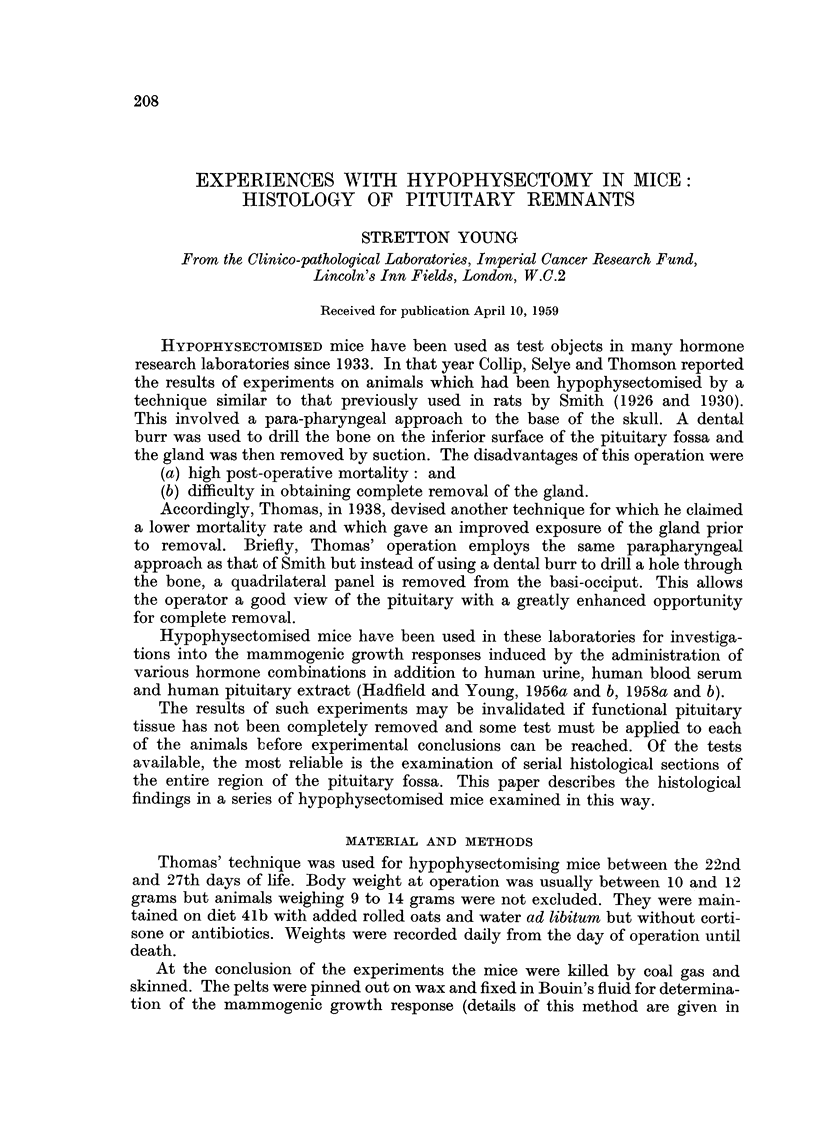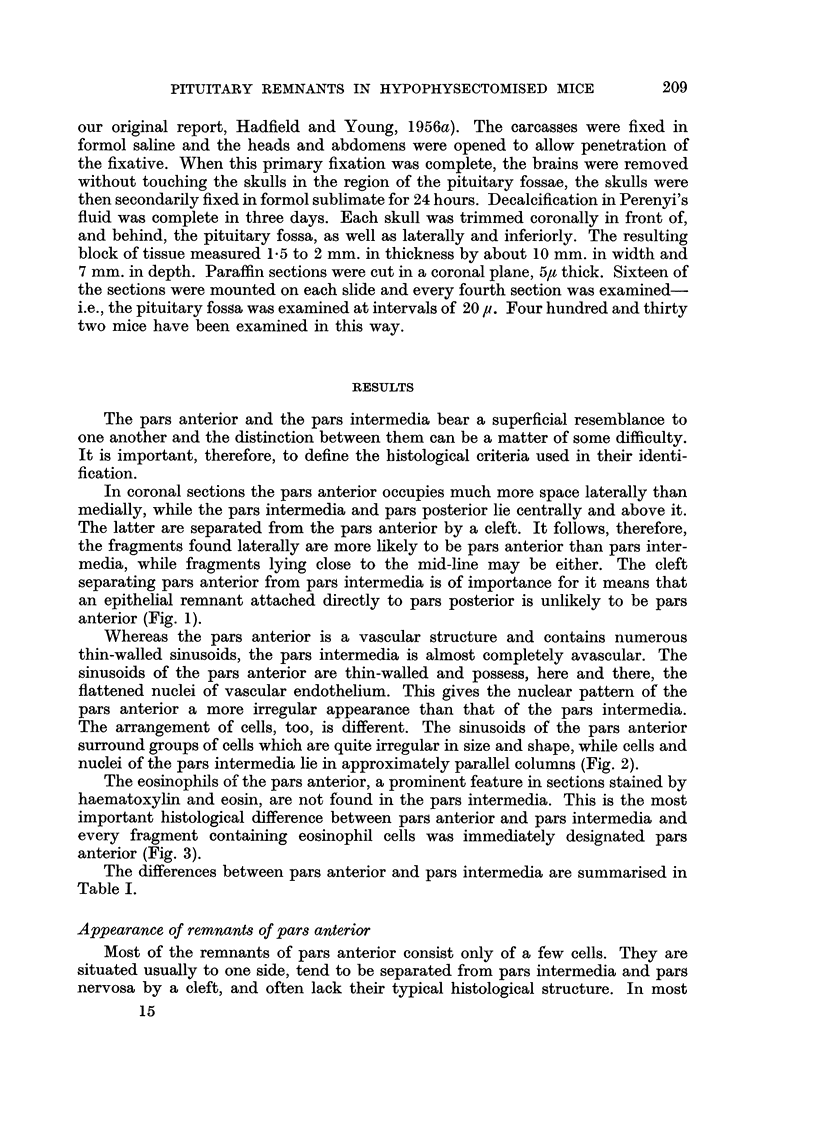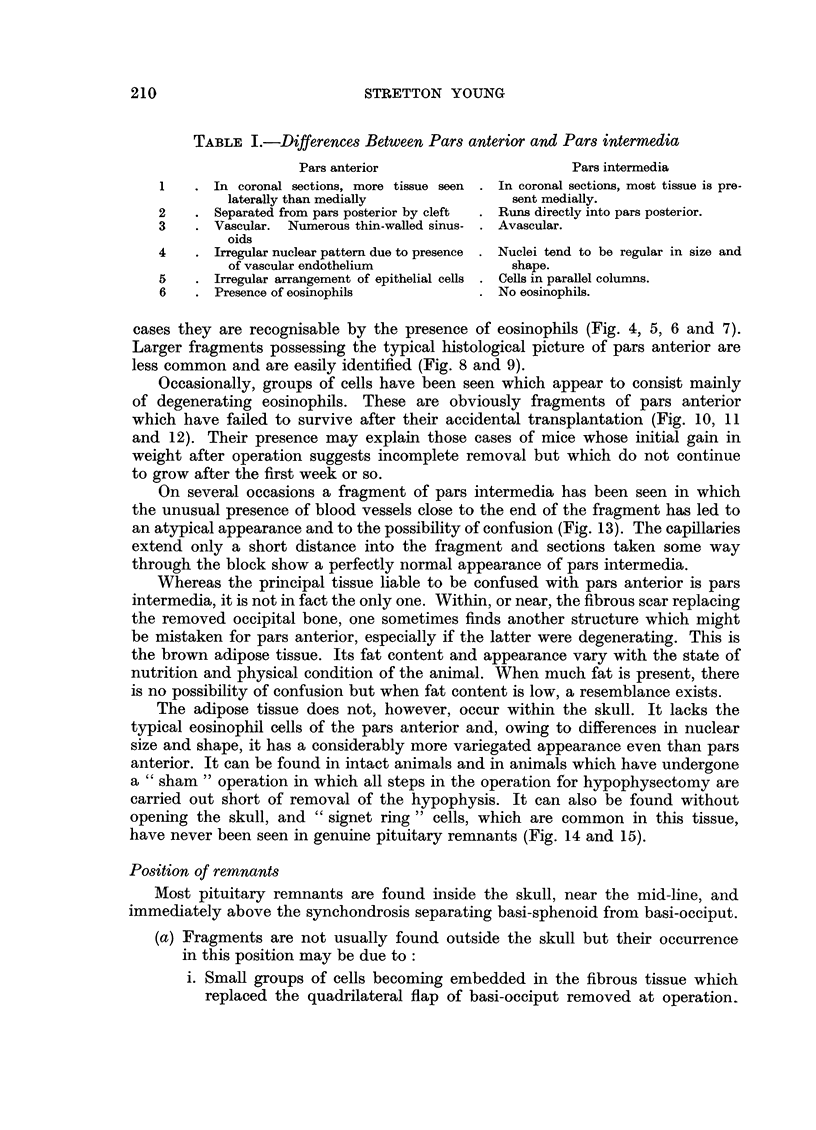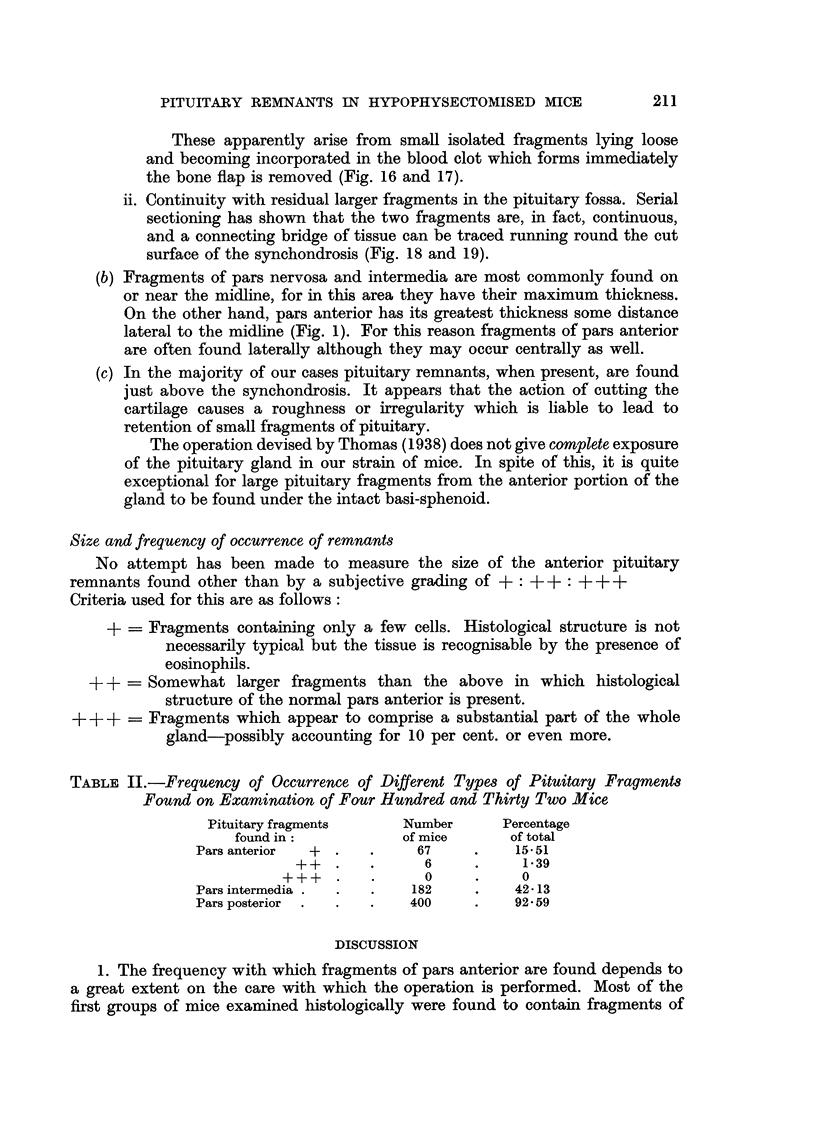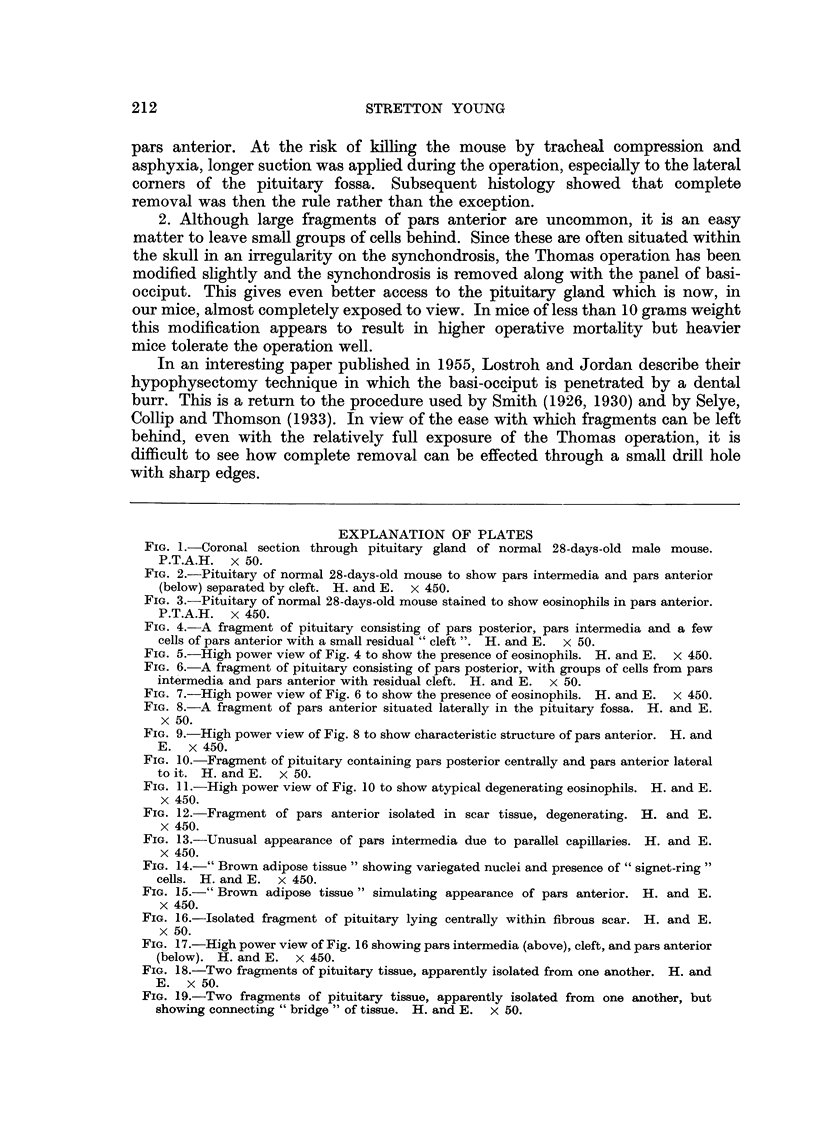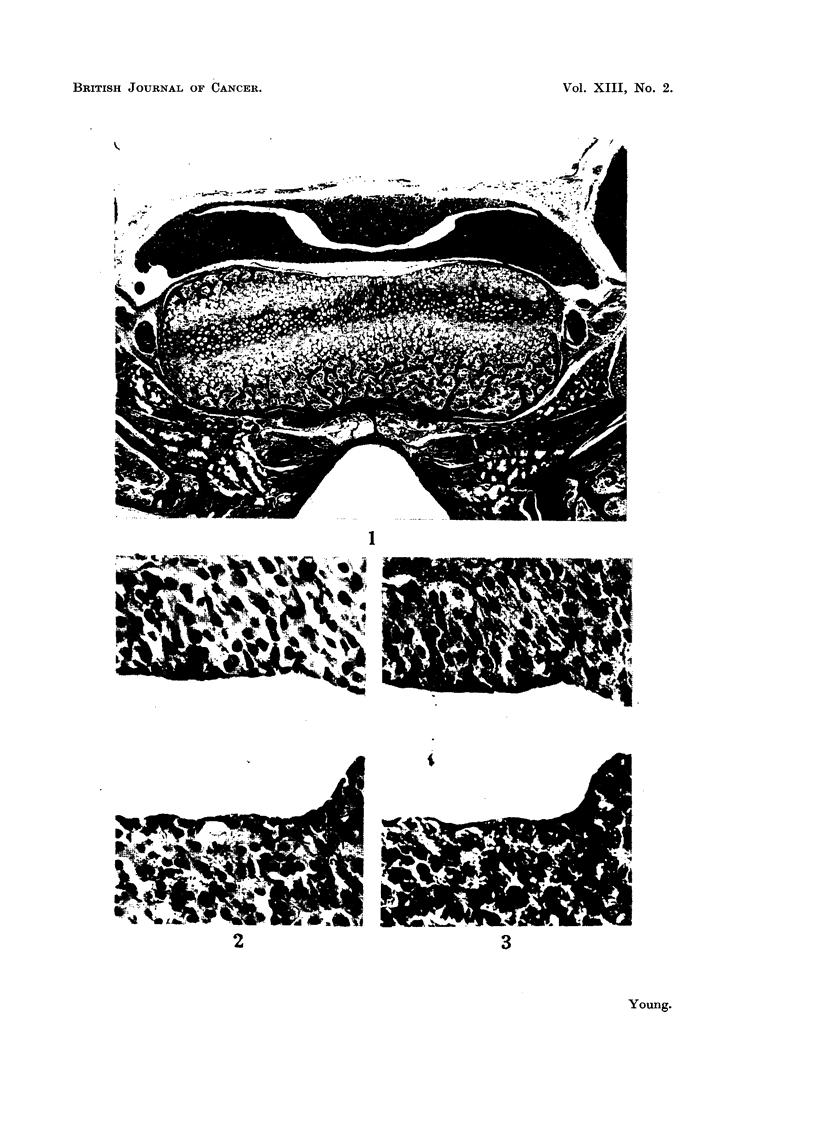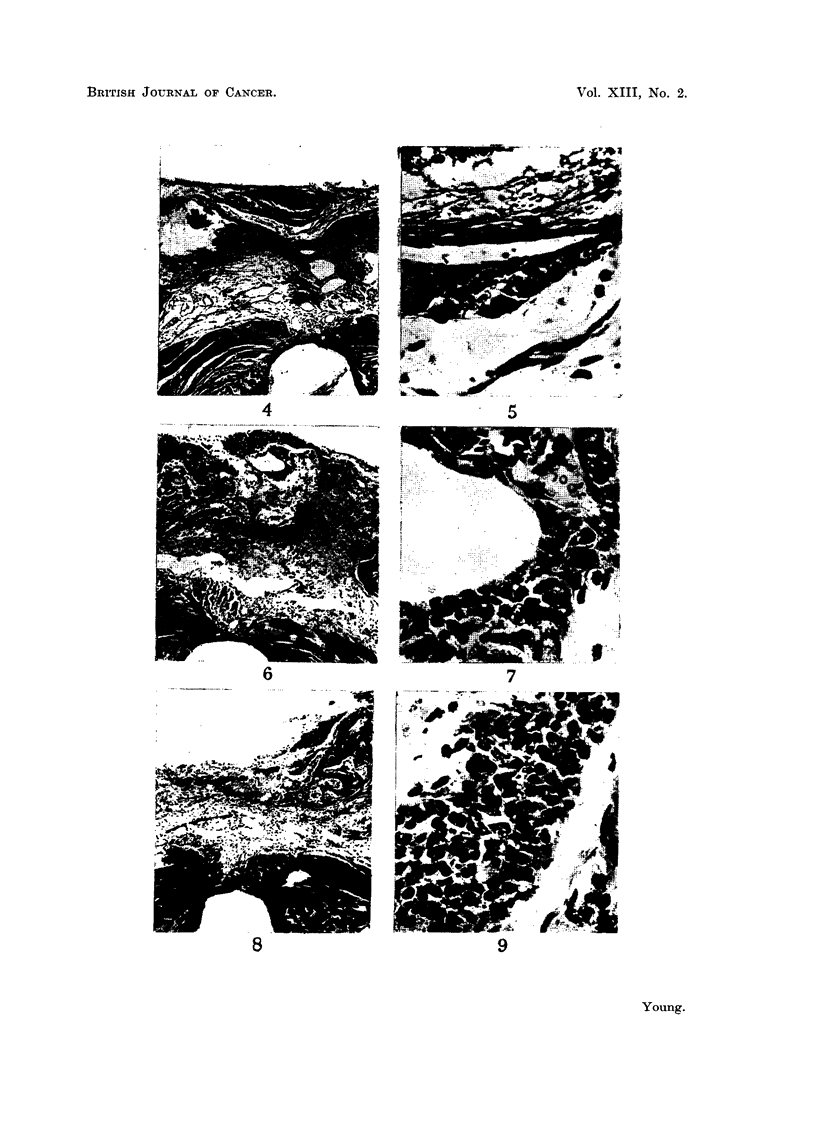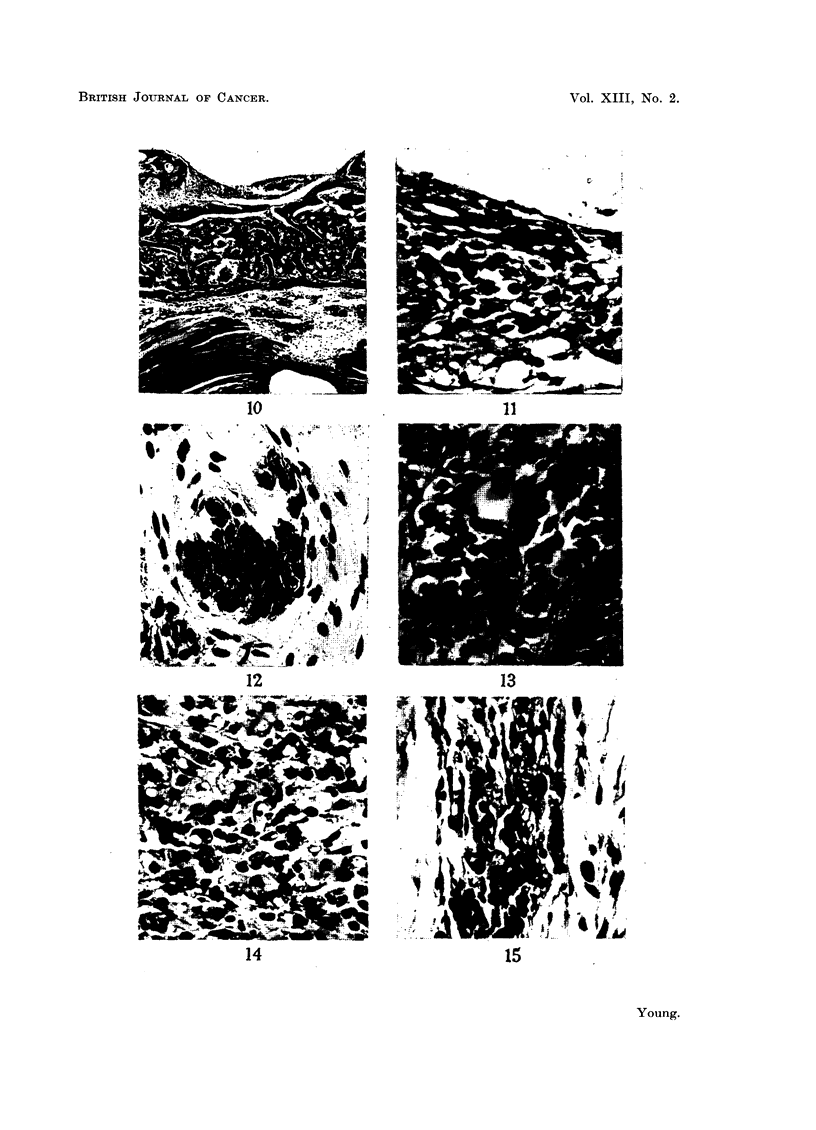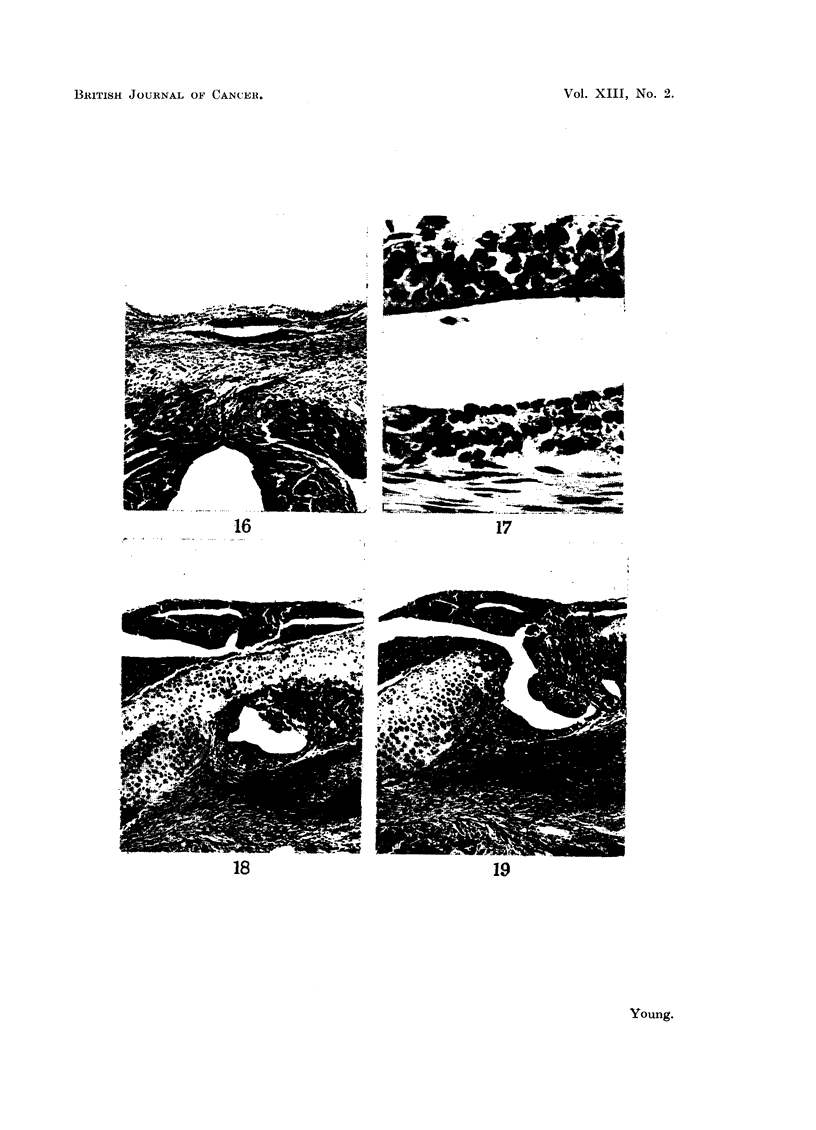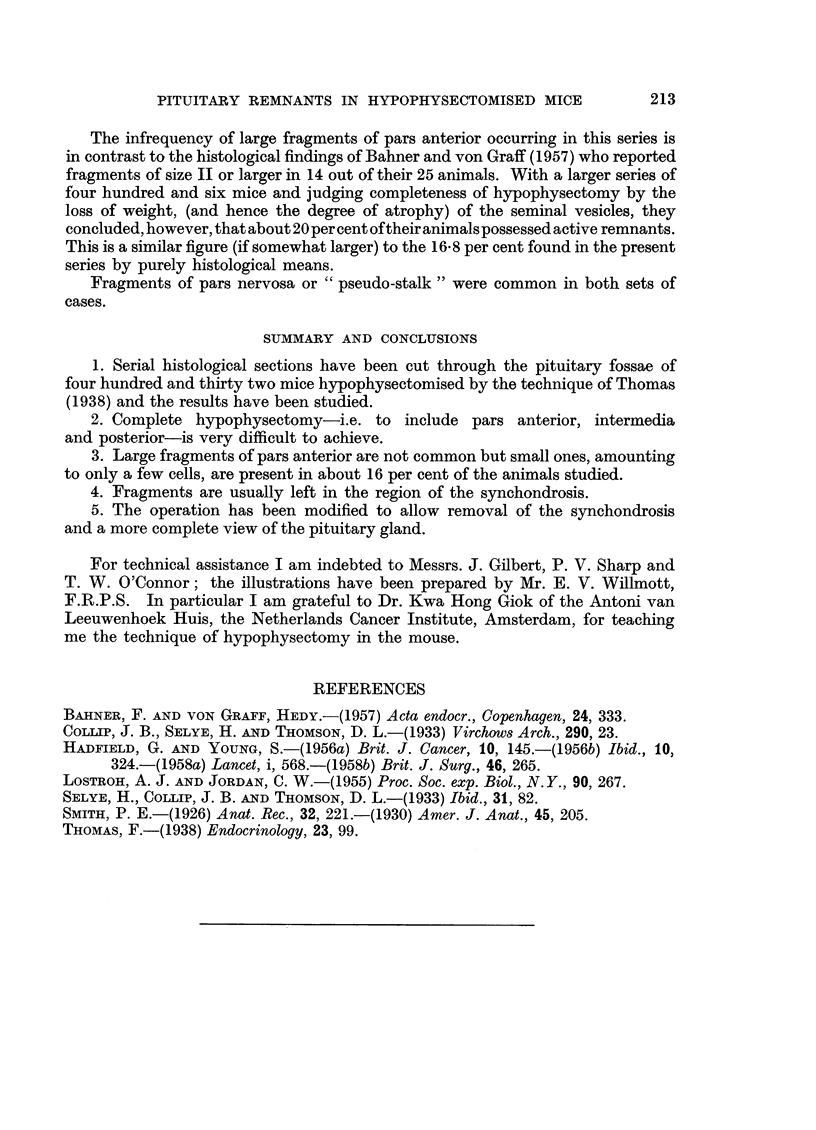# Experiences with Hypophysectomy in Mice: Histology of Pituitary Remnants

**DOI:** 10.1038/bjc.1959.28

**Published:** 1959-06

**Authors:** Stretton Young

## Abstract

**Images:**


### EXPERIENCES WITH HYPOPHYSECTOMY IN MICE: HISTOLOGY OF PITUITARY REMNANTS

STRETTON YOUNG

*From the Clinico-pathological Laboratories, Imperial Cancer Research Fund, Lincoln's Inn Fields, London, W.C.2*

Received for publication April 10, 1959

HYPOPHYSECTOMISED mice have been used as test objects in many hormone research laboratories since 1933. In that year Collip, Selye and Thomson reported the results of experiments on animals which had been hypophysectomised by a technique similar to that previously used in rats by Smith (1926 and 1930). This involved a para-pharyngeal approach to the base of the skull. A dental burr was used to drill the bone on the inferior surface of the pituitary fossa and the gland was then removed by suction. The disadvantages of this operation were

(*a*) high post-operative mortality : and

(*b*) difficulty in obtaining complete removal of the gland.

Accordingly, Thomas, in 1938, devised another technique for which he claimed a lower mortality rate and which gave an improved exposure of the gland prior to removal. Briefly, Thomas' operation employs the same parapharyngeal approach as that of Smith but instead of using a dental burr to drill a hole through the bone, a quadrilateral panel is removed from the basi-occiput. This allows the operator a good view of the pituitary with a greatly enhanced opportunity for complete removal.

Hypophysectomised mice have been used in these laboratories for investigations into the mammogenic growth responses induced by the administration of various hormone combinations in addition to human urine, human blood serum and human pituitary extract (Hadfield and Young, 1956*a* and *b*, 1958*a* and *b*).

The results of such experiments may be invalidated if functional pituitary tissue has not been completely removed and some test must be applied to each of the animals before experimental conclusions can be reached. Of the tests available, the most reliable is the examination of serial histological sections of the entire region of the pituitary fossa. This paper describes the histological findings in a series of hypophysectomised mice examined in this way.

### MATERIAL AND METHODS

Thomas' technique was used for hypophysectomising mice between the 22nd and 27th days of life. Body weight at operation was usually between 10 and 12 grams but animals weighing 9 to 14 grams were not excluded. They were maintained on diet 41b with added rolled oats and water *ad libitum* but without cortisone or antibiotics. Weights were recorded daily from the day of operation until death.

At the conclusion of the experiments the mice were killed by coal gas and skinned. The pelts were pinned out on wax and fixed in Bouin's fluid for determination of the mammogenic growth response (details of this method are given in our original report, Hadfield and Young, 1956*a*). The carcasses were fixed in formol saline and the heads and abdomens were opened to allow penetration of the fixative. When this primary fixation was complete, the brains were removed without touching the skulls in the region of the pituitary fossae, the skulls were then secondarily fixed in formol sublimate for 24 hours. Decalcification in Perenyi's fluid was complete in three days. Each skull was trimmed coronally in front of, and behind, the pituitary fossa, as well as laterally and inferiorly. The resulting block of tissue measured 1·5 to 2 mm. in thickness by about 10 mm. in width and 7 mm. in depth. Paraffin sections were cut in a coronal plane, 5$\mu$ thick. Sixteen of the sections were mounted on each slide and every fourth section was examined—i.e., the pituitary fossa was examined at intervals of 20 $\mu$. Four hundred and thirty two mice have been examined in this way.

### RESULTS

The pars anterior and the pars intermedia bear a superficial resemblance to one another and the distinction between them can be a matter of some difficulty. It is important, therefore, to define the histological criteria used in their identification.

In coronal sections the pars anterior occupies much more space laterally than medially, while the pars intermedia and pars posterior lie centrally and above it. The latter are separated from the pars anterior by a cleft. It follows, therefore, the fragments found laterally are more likely to be pars anterior than pars intermedia, while fragments lying close to the mid-line may be either. The cleft separating pars anterior from pars intermedia is of importance for it means that an epithelial remnant attached directly to pars posterior is unlikely to be pars anterior (Fig. 1).

Whereas the pars anterior is a vascular structure and contains numerous thin-walled sinusoids, the pars intermedia is almost completely avascular. The sinusoids of the pars anterior are thin-walled and possess, here and there, the flattened nuclei of vascular endothelium. This gives the nuclear pattern of the pars anterior a more irregular appearance than that of the pars intermedia. The arrangement of cells, too, is different. The sinusoids of the pars anterior surround groups of cells which are quite irregular in size and shape, while cells and nuclei of the pars intermedia lie in approximately parallel columns (Fig. 2).

The eosinophils of the pars anterior, a prominent feature in sections stained by haematoxylin and eosin, are not found in the pars intermedia. This is the most important histological difference between pars anterior and pars intermedia and every fragment containing eosinophil cells was immediately designated pars anterior (Fig. 3).

The differences between pars anterior and pars intermedia are summarised in Table I.

### *Appearance of remnants of pars anterior*

Most of the remnants of pars anterior consist only of a few cells. They are situated usually to one side, tend to be separated from pars intermedia and pars nervosa by a cleft, and often lack their typical histological structure. In most

TABLE I.—*Differences Between Pars anterior and Pars intermedia*

| | Pars anterior | Pars intermedia |
|---|---|---|
| 1 | In coronal sections, more tissue seen laterally than medially | In coronal sections, most tissue is present medially. |
| 2 | Separated from pars posterior by cleft | Runs directly into pars posterior. |
| 3 | Vascular. Numerous thin-walled sinusoids | Avascular. |
| 4 | Irregular nuclear pattern due to presence of vascular endothelium | Nuclei tend to be regular in size and shape. |
| 5 | Irregular arrangement of epithelial cells | Cells in parallel columns. |
| 6 | Presence of eosinophils | No eosinophils. |

cases they are recognisable by the presence of eosinophils (Fig. 4, 5, 6 and 7). Larger fragments possessing the typical histological picture of pars anterior are less common and are easily identified (Fig. 8 and 9).

Occasionally, groups of cells have been seen which appear to consist mainly of degenerating eosinophils. These are obviously fragments of pars anterior which have failed to survive after their accidental transplantation (Fig. 10, 11 and 12). Their presence may explain those cases of mice whose initial gain in weight after operation suggests incomplete removal but which do not continue to grow after the first week or so.

On several occasions a fragment of pars intermedia has been seen in which the unusual presence of blood vessels close to the end of the fragment has led to an atypical appearance and to the possibility of confusion (Fig. 13). The capillaries extend only a short distance into the fragment and sections taken some way through the block show a perfectly normal appearance of pars intermedia.

Whereas the principal tissue liable to be confused with pars anterior is pars intermedia, it is not in fact the only one. Within, or near, the fibrous scar replacing the removed occipital bone, one sometimes finds another structure which might be mistaken for pars anterior, especially if the latter were degenerating. This is the brown adipose tissue. Its fat content and appearance vary with the state of nutrition and physical condition of the animal. When much fat is present, there is no possibility of confusion but when fat content is low, a resemblance exists.

The adipose tissue does not, however, occur within the skull. It lacks the typical eosinophil cells of the pars anterior and, owing to differences in nuclear size and shape, it has a considerably more variegated appearance even than pars anterior. It can be found in intact animals and in animals which have undergone a " sham " operation in which all steps in the operation for hypophysectomy are carried out short of removal of the hypophysis. It can also be found without opening the skull, and " signet ring " cells, which are common in this tissue, have never been seen in genuine pituitary remnants (Fig. 14 and 15).

*Position of remnants*

Most pituitary remnants are found inside the skull, near the mid-line, and immediately above the synchondrosis separating basi-sphenoid from basi-occiput.

(*a*) Fragments are not usually found outside the skull but their occurrence in this position may be due to :

i. Small groups of cells becoming embedded in the fibrous tissue which replaced the quadrilateral flap of basi-occiput removed at operation.

These apparently arise from small isolated fragments lying loose and becoming incorporated in the blood clot which forms immediately the bone flap is removed (Fig. 16 and 17).

ii. Continuity with residual larger fragments in the pituitary fossa. Serial sectioning has shown that the two fragments are, in fact, continuous, and a connecting bridge of tissue can be traced running round the cut surface of the synchondrosis (Fig. 18 and 19).

(*b*) Fragments of pars nervosa and intermedia are most commonly found on or near the midline, for in this area they have their maximum thickness. On the other hand, pars anterior has its greatest thickness some distance lateral to the midline (Fig. 1). For this reason fragments of pars anterior are often found laterally although they may occur centrally as well.

(*c*) In the majority of our cases pituitary remnants, when present, are found just above the synchondrosis. It appears that the action of cutting the cartilage causes a roughness or irregularity which is liable to lead to retention of small fragments of pituitary.

The operation devised by Thomas (1938) does not give *complete* exposure of the pituitary gland in our strain of mice. In spite of this, it is quite exceptional for large pituitary fragments from the anterior portion of the gland to be found under the intact basi-sphenoid.

*Size and frequency of occurrence of remnants*

No attempt has been made to measure the size of the anterior pituitary remnants found other than by a subjective grading of + : ++ : +++ Criteria used for this are as follows :

+ = Fragments containing only a few cells. Histological structure is not necessarily typical but the tissue is recognisable by the presence of eosinophils.

++ = Somewhat larger fragments than the above in which histological structure of the normal pars anterior is present.

+++ = Fragments which appear to comprise a substantial part of the whole gland—possibly accounting for 10 per cent. or even more.

TABLE II.—*Frequency of Occurrence of Different Types of Pituitary Fragments Found on Examination of Four Hundred and Thirty Two Mice*

| Pituitary fragments found in : | | Number of mice | Percentage of total |
|---|---|---|---|
| Pars anterior | + | 67 | 15·51 |
| | ++ | 6 | 1·39 |
| | +++ | 0 | 0 |
| Pars intermedia | | 182 | 42·13 |
| Pars posterior | | 400 | 92·59 |

DISCUSSION

1. The frequency with which fragments of pars anterior are found depends to a great extent on the care with which the operation is performed. Most of the first groups of mice examined histologically were found to contain fragments of pars anterior. At the risk of killing the mouse by tracheal compression and asphyxia, longer suction was applied during the operation, especially to the lateral corners of the pituitary fossa. Subsequent histology showed that complete removal was then the rule rather than the exception.

2. Although large fragments of pars anterior are uncommon, it is an easy matter to leave small groups of cells behind. Since these are often situated within the skull in an irregularity on the synchondrosis, the Thomas operation has been modified slightly and the synchondrosis is removed along with the panel of basi-occiput. This gives even better access to the pituitary gland which is now, in our mice, almost completely exposed to view. In mice of less than 10 grams weight this modification appears to result in higher operative mortality but heavier mice tolerate the operation well.

In an interesting paper published in 1955, Lostroh and Jordan describe their hypophysectomy technique in which the basi-occiput is penetrated by a dental burr. This is a return to the procedure used by Smith (1926, 1930) and by Selye, Collip and Thomson (1933). In view of the ease with which fragments can be left behind, even with the relatively full exposure of the Thomas operation, it is difficult to see how complete removal can be effected through a small drill hole with sharp edges.

---

### EXPLANATION OF PLATES

FIG. 1.—Coronal section through pituitary gland of normal 28-days-old male mouse. P.T.A.H. × 50.

FIG. 2.—Pituitary of normal 28-days-old mouse to show pars intermedia and pars anterior (below) separated by cleft. H. and E. × 450.

FIG. 3.—Pituitary of normal 28-days-old mouse stained to show eosinophils in pars anterior. P.T.A.H. × 450.

FIG. 4.—A fragment of pituitary consisting of pars posterior, pars intermedia and a few cells of pars anterior with a small residual " cleft ". H. and E. × 50.

FIG. 5.—High power view of Fig. 4 to show the presence of eosinophils. H. and E. × 450.

FIG. 6.—A fragment of pituitary consisting of pars posterior, with groups of cells from pars intermedia and pars anterior with residual cleft. H. and E. × 50.

FIG. 7.—High power view of Fig. 6 to show the presence of eosinophils. H. and E. × 450.

FIG. 8.—A fragment of pars anterior situated laterally in the pituitary fossa. H. and E. × 50.

FIG. 9.—High power view of Fig. 8 to show characteristic structure of pars anterior. H. and E. × 450.

FIG. 10.—Fragment of pituitary containing pars posterior centrally and pars anterior lateral to it. H. and E. × 50.

FIG. 11.—High power view of Fig. 10 to show atypical degenerating eosinophils. H. and E. × 450.

FIG. 12.—Fragment of pars anterior isolated in scar tissue, degenerating. H. and E. × 450.

FIG. 13.—Unusual appearance of pars intermedia due to parallel capillaries. H. and E. × 450.

FIG. 14.—" Brown adipose tissue " showing variegated nuclei and presence of " signet-ring " cells. H. and E. × 450.

FIG. 15.—" Brown adipose tissue " simulating appearance of pars anterior. H. and E. × 450.

FIG. 16.—Isolated fragment of pituitary lying centrally within fibrous scar. H. and E. × 50.

FIG. 17.—High power view of Fig. 16 showing pars intermedia (above), cleft, and pars anterior (below). H. and E. × 450.

FIG. 18.—Two fragments of pituitary tissue, apparently isolated from one another. H. and E. × 50.

FIG. 19.—Two fragments of pituitary tissue, apparently isolated from one another, but showing connecting " bridge " of tissue. H. and E. × 50.

1

2

3

Young.

4

5

6

7

8

9

Young.

10

11

12

13

14

15

Young.

16

17

18

19

The infrequency of large fragments of pars anterior occurring in this series is in contrast to the histological findings of Bahner and von Graff (1957) who reported fragments of size II or larger in 14 out of their 25 animals. With a larger series of four hundred and six mice and judging completeness of hypophysectomy by the loss of weight, (and hence the degree of atrophy) of the seminal vesicles, they concluded, however, that about 20 per cent of their animals possessed active remnants. This is a similar figure (if somewhat larger) to the 16·8 per cent found in the present series by purely histological means.

Fragments of pars nervosa or " pseudo-stalk " were common in both sets of cases.

### SUMMARY AND CONCLUSIONS

1. Serial histological sections have been cut through the pituitary fossae of four hundred and thirty two mice hypophysectomised by the technique of Thomas (1938) and the results have been studied.
2. Complete hypophysectomy—i.e. to include pars anterior, intermedia and posterior—is very difficult to achieve.
3. Large fragments of pars anterior are not common but small ones, amounting to only a few cells, are present in about 16 per cent of the animals studied.
4. Fragments are usually left in the region of the synchondrosis.
5. The operation has been modified to allow removal of the synchondrosis and a more complete view of the pituitary gland.

For technical assistance I am indebted to Messrs. J. Gilbert, P. V. Sharp and T. W. O'Connor ; the illustrations have been prepared by Mr. E. V. Willmott, F.R.P.S. In particular I am grateful to Dr. Kwa Hong Giok of the Antoni van Leeuwenhoek Huis, the Netherlands Cancer Institute, Amsterdam, for teaching me the technique of hypophysectomy in the mouse.

### REFERENCES

Bahner, F. and von Graff, Hedy.—(1957) *Acta endocr., Copenhagen,* **24**, 333.
Collip, J. B., Selye, H. and Thomson, D. L.—(1933) *Virchows Arch.*, **290**, 23.
Hadfield, G. and Young, S.—(1956*a*) *Brit. J. Cancer,* **10**, 145.—(1956*b*) *Ibid.*, **10**, 324.—(1958*a*) *Lancet*, i, 568.—(1958*b*) *Brit. J. Surg.*, **46**, 265.
Lostroh, A. J. and Jordan, C. W.—(1955) *Proc. Soc. exp. Biol., N.Y.*, **90**, 267.
Selye, H., Collip, J. B. and Thomson, D. L.—(1933) *Ibid.*, **31**, 82.
Smith, P. E.—(1926) *Anat. Rec.*, **32**, 221.—(1930) *Amer. J. Anat.*, **45**, 205.
Thomas, F.—(1938) *Endocrinology,* **23**, 99.